# Expression Patterns of Key Genes in the Photoperiod and Vernalization Flowering Pathways in *Lilium longiflorum* with Different Bulb Sizes

**DOI:** 10.3390/ijms23158341

**Published:** 2022-07-28

**Authors:** Xiao Yan, Lian-Juan Wang, Yu-Qian Zhao, Gui-Xia Jia

**Affiliations:** Beijing Key Laboratory of Ornamental Plants Germplasm Innovation & Molecular Breeding, National Engineering Research Center for Floriculture, Beijing Laboratory of Urban and Rural Ecological Environment, Key Laboratory of Genetics and Breeding in Forest Trees and Ornamental Plants of Ministry of Education, School of Landscape Architecture, Beijing Forestry University, Beijing 100083, China; yanxiao102109@163.com (X.Y.); wlj12523@163.com (L.-J.W.); zhaoyuq1992@163.com (Y.-Q.Z.)

**Keywords:** bulb size, flowering related genes, *Lilium longiflorum*, photoperiod, vernalization

## Abstract

*Lilium longiflorum* is a wild *Lilium*, and its flowering transition requires a long period of cold exposure to meet the demand of vernalization. The responses of different sized bulbs to cold exposure and photoperiod are different, and the floral transition pathways of small and large bulbs are different. In this study, small and large bulbs were placed in cold storage for different weeks and then cultured at a constant ambient temperature of 25 °C under long day (LD) and short day (SD) conditions. Then, the flowering characteristics and expression patterns of key genes related to the vernalization and photoperiod pathways in different groups were calculated and analyzed. The results showed that the floral transition of *Lilium longiflorum* was influenced by both vernalization and photoperiod, that vernalization and LD conditions can significantly improve the flowering rate of *Lilium longiflorum*, and that the time from planting to visible flowering buds’ appearance was decreased. The flowering time and rate of large bulbs were greatly influenced by cold exposure, and the vernalization pathway acted more actively at the floral transition stage. The floral transition of small bulbs was affected more by the photoperiod pathway. Moreover, it was speculated that cold exposure may promote greater sensitivity of the small bulbs to LD conditions. In addition, the expression of *LlVRN1*, *LlFKF1*, *LlGI*, *LlCO5*, *LlCO7*, *LlCO16*, *LlFT1*, *LlFT3* and *LlSOC1* was high during the process of floral transition, and *LlCO13*, *LlCO14* and *LlCO15* were highly expressed in the vegetative stage. The expression of *LlCO13* and *LlCO14* was different under different lighting conditions, and the flowering induction function of *LlCO9* and *LlFT3* was related to vernalization. Moreover, *LlFKF1*, *LlGI*, *LlCO5*, *LlCO16*, *LlSOC1* and *LlFT2* were involved in the entire growth process of plants, while *LlCO6*, *LlCO16* and *LlFT1* are involved in the differentiation and formation of small bulblets of plants after the inflorescence stage, and this process is also closely related to LD conditions. This study has great significance for understanding the molecular mechanisms of the vernalization and photoperiod flowering pathways of *Lilium longiflorum*.

## 1. Introduction

Flowering is essential to most plants, and the flowering process is regulated by environmental and internal signals [1]. At present, five classic flowering pathways have been identified in *Arabidopsis*, among which vernalization and photoperiod pathways have been studied extensively. In *Arabidopsis*, the *FLOWERING LOCUS T* (*FT*) gene is the key regulator of flowering time [2,3], and the *FT* gene regulates flowering time by integrating both environmental and endogenous signals from multiple pathways. The *FT* gene belongs to the *FT/TFL1*(*TERMINAL FLOWER1*) gene family. The FT protein is synthesized in the leaves and then transported to the shoot apical meristem (SAM). It interacts with the b-ZIP transcription factor FLOWERING LOCUS D(FD) to form FT/FD protein complexes in the SAM [4], after which it induces flowering by promoting the expression of *SUPPERSSOR OF OVEREXPRESSION OF CONSTANS1*(*SOC1*), *APETALA1*(*AP1*), *FRUITFULL*(*FUL*) and *LEAFY* (*LFY*) [5]. To date, the *FT* gene family has been found in many plants, and the numbers and functions of *FT* genes vary in different plants [6,7,8,9,10,11,12,13]. Yan et al. (2021) identified three *Lilium FLOWERING LOCUS T* (*LFT*) family members, and *LFT1* and *LFT3* were highly expressed at the floral transition stage and may promote floral induction, while *LFT2* may be involved in the differentiation of bulblets [14].

*FLOWERING LOCUS C* (*FLC*) is the most studied and the most important gene in the vernalization pathway; it encodes a potent MAD-box gene that inhibits blooming in *Arabidopsis*. *FLC* can directly inhibit or induce the *FRIGIDA* (*FRI*) gene to inhibit the expression of floral meristem formation genes. The expression of *FT* was inhibited by *FLC* at the beginning of winter, and the expression of *FLC* was downregulated after a period of vernalization [15]. The genes *VERNALIZATION* *1*(*VRN1*) and *VERNALIZATION 2* (*VRN2*) that encode zinc-finger proteins are also key genes for vernalization in *Arabidopsis*, and they can promote flowering by continuously inhibiting *FLC* expression [16,17]. In wheat, the flowering repressor is the VRN2 protein, and *VRN1* is one of the genes inhibited by *VRN2*. *VRN1* encodes a protein that promotes plant flowering [18,19]. In cereals, the *VRN1* gene plays a crucial and dual role in the process of flowering; it can not only induce the expression of *FT* homologues by vernalization, but can also be used as a floral meristem identity gene [19,20]. Liu et al. (2014) identified *LoVRN1*, which can promote the early flowering and change the flower type in *Arabidopsis*, in the Oriental lily ‘Sorbonne’ [21].

In *Arabidopsis*, the *CONSTANS*/*FLOWERING LOCUS T* (*CO/FT*) module plays a central role in the photoperiodic flowering pathway [22]. In the photoperiodic regulation of flowering, *GIGANTEA* (*GI*) and *FLAVIN-BINDING KELCH REPEAT F-BOX 1* (*FKF1*) respond to changes in light length and regulate the expression of *CO* [23,24]. The *CO* gene regulates flowering time by promoting transcription and affecting the expression of the downstream genes *SOC1* and *FT* [5]. The *CONSTANS/CONSTANS-LIKE* (*CO/COL*) family members play a very important role in the *CO-FT* pathway [25,26]. COL proteins generally contain two conserved domains:the B-box(BBX) and CCT (CO, CO-LIKE and TOC1) structural domains [27]. CO/COL regulates flowering both in *Arabidopsis* and rice [28], and its homologous gene sequence and function are highly conserved and present in many varieties of plants [29]. Most of the *COL* genes are similar in structure during plant evolution, and all of them are regulated by circadian rhythms and photoperiods, but some of them also have different functions [28]. For instance, in addition to playing a negative regulatory role in the photoperiod, *AtCOL3* also promotes the development of lateral roots [30], and *AtCOL7* promotes stem branching in environments with a high ratio of red light/far-red light [31]. Recent studies have identified many CO homologues in other plants, such as *PhalCOL* in *Phalaenopsis hybrida*, *MaCOL1* in banana, *StCO* in potato, *VvCOL1* and *VvCOL5* in grape, *PbCOL8* in pear, and *NnCOL5* in *Nelumbo nucifera* [28,32,33,34,35,36,37,38].

*Lilium longiflorum* belongs to Liliaceae, and is a horticultural plant [39]. Flowering of plants is the key to their successful reproduction, and many plants need to go through a cold phase to break dormancy to bloom [40]. Studies have shown that the flowering transition of *Lilium* *longiflorum* requires a long period of cold exposure to meet the demand of vernalization. Vernalization is a necessary condition for the flowering of *Lilium longiflorum*, and it is also the main factor that affects the flowering period and quality [41]. Bulbs of *Lilium* *longiflorum* (cultivar ‘White Heaven’) grown under a constant temperature of 25 °C without vernalization formed only leaves and did not bloom for more than 15 months, which proved the necessity of vernalization of this cultivar [42]. During the entire growth process of the plant, from the cold exposure stage to flower initiation, all stages have the ability to induce the vernalization signal [43]. The study of morphological and biochemical markers showed that cold storage of bulbs can promote flowering [44]. In *Lilium* *longiflorum* ‘White Heaven’, cold exposure greatly promoted stem elongation and shortened the time from planting to flowering [42,45]. However, when the bulb sizes are different and under certain conditions or during special periods of development, the LD photoperiod can promote the flowering transition of lily and may replace cold storage as the factor inducing flower formation [41,43,46]. The latest research shows that small bulbs of nonvernalized *Lilium* *longiflorum* can bloom under long-day (LD) conditions, indicating that small bulbs of *Lilium longiflorum* flowered by the photoperiodic pathway, rather than vernalization [46]. However, the key genes involved in vernalization and photoperiod flowering pathways and the developmental stages at which they act warrant further studies.

In geophytes, such as lilies, tulips, lycoris, irises and onions, the size of the bulbs during planting affects their subsequent growth and development as well as their induction by vernalization and florogenesis [47,48,49,50]. When the bulb reaches the critical size required for flowering transition, the quality of flowering is usually positively correlated with bulb size [51]. The heavier the bulb mass is, the greater the ability of *Lilium oriental* and *Leucocoryne* to produce superior flowers [52]. At present, studies on lily bulbs of different sizes mainly focus on morphology, starch metabolism, soluble sugar and endogenous hormones [46,53,54,55,56], and there are no reports on the effects of bulb size on flowering nor on the underlying molecular mechanisms. According to the research group’s study, eight *LfCOL* genes were obtained from lily, the *LfCOL5*, *LfCOL6* and *LfCOL9* genes participate in flowering induction under long days, and the overexpression of those genes rescued the late flowering phenotype of the *co*-1 mutant in *Arabidopsis* [13]. Therefore, to elucidate the molecular mechanism of flowering under different conditions for different bulb sizes of *Lilium longiflorum* and to lay a foundation for exploring the relationship among lily bulb size, cold exposure time and photoperiod, the flowering characteristics and the expression patterns of key genes in the vernalization and photoperiod pathways were analysed in *Lilium longiflorum*.

## 2. Results

### 2.1. Flowering Characteristics of Lilium Longiflorum under Different Conditions

**Figure 1:**
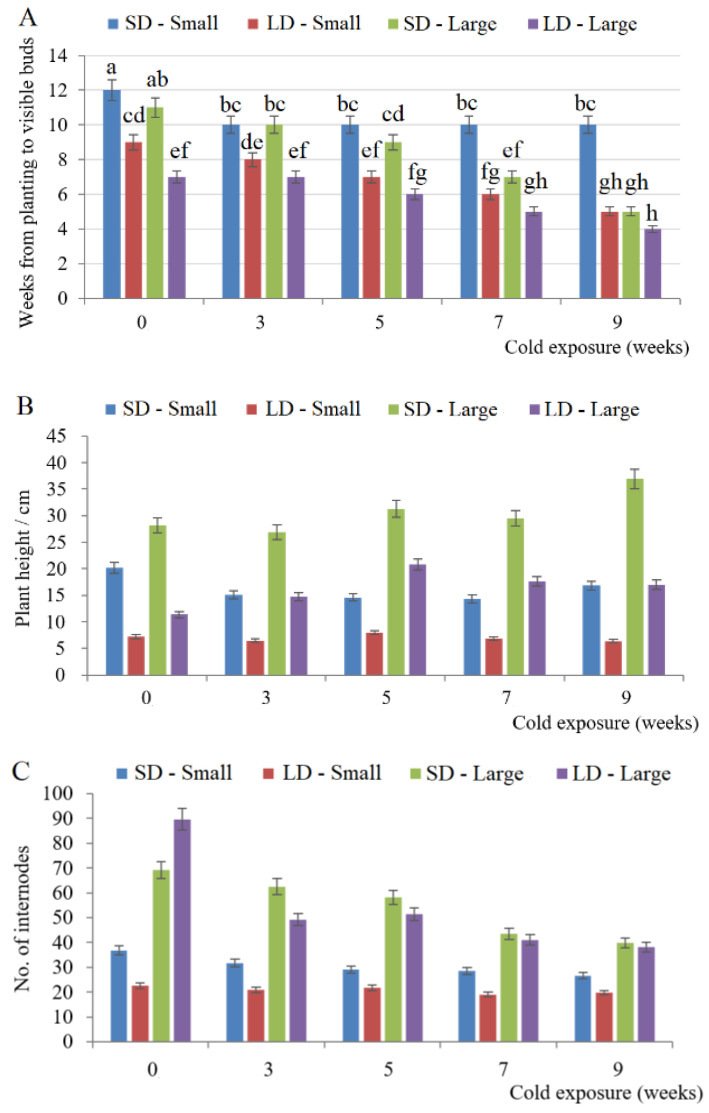

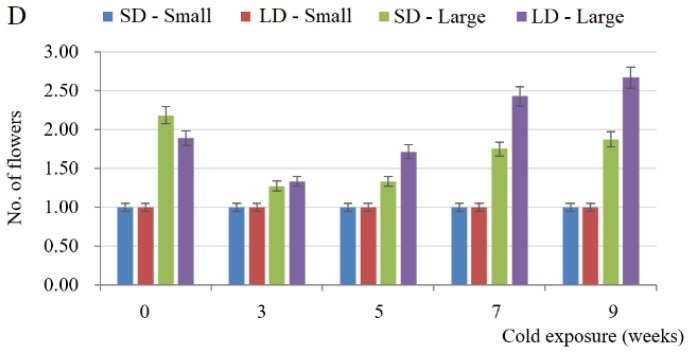
Effect of cold exposure and photoperiod on the flowering time and morphological characteristics of different sized bulbs of *Lilium longiflorum*. (**A**) Time from planting to visible flower bud appearance. (**B**) Plant height at visible bud appearance stage. (**C**) Number of internodes at visible bud appearance stage. (**D**) Average number of flowers. Notes: SD, short day (light/dark: 8/16 h); LD, long day (light/dark: 16/8 h); Small and large, bulb size; 0–9, weeks of bulb exposure to 4 °C before planting. Different lowercase letters indicate significant differences (*p* < 0.05).

The bulbs of all treatment groups were planted on the same day and the plants in all treatment groups emerged one week after planting. The flowering time and rate of different groups were statistically analyzed (plants that did not flower remained in the vegetative growth stage) (Figure 1, Table 1). For the small bulbs without vernalization grown under SD conditions, the time from planting to visible flowering bud (VB) was 12 weeks. The time from planting to VB of the 3-, 5-, 7- and 9-week cold exposure treatment groups was 10 weeks (Figure 1A). Under SD conditions, with increasing cold storage time, the flowering rate of small bulbs increased from 10% to 42.1% (Table 1). For the small bulbs planted under LD conditions, when the time of cold-storage increased, the time from planting to VB appearance decreased from 9 weeks to 5 weeks (Figure 1A), and the flowering rate increased from 52.1% to 93.25%. Therefore, compared with SD conditions, LD conditions increased the flowering rate of nonvernalized small bulbs by 42.1% (52.1−10%) and the 9 weeks cold-exposed small bulbs by 51.15% (93.25−42.1%); moreover, compared with the nonvernalized small bulbs, 9 weeks of cold exposure increased the flowering rates of small bulbs by 32.1% (42.1−10%) under SD and 41.15% (93.25−52.1%) under LD conditions (Table 1).

For the small bulbs, the combination of LD and vernalization can significantly improve the flowering rate, and LD conditions played an important role in the flowering of both nonvernalized and cold-exposed small bulbs (Table 1). For the small bulbs planted under LD conditions, the time required for flowering was negatively correlated with the length of cold-storage time (Figure 1A). Notably, the time from planting to visible flowering bud (VB) of the small bulbs without vernalization grown under LD conditions was 9 weeks, and the time from planting to VB was 10 weeks in the 3-, 5-, 7- and 9-week cold exposure groups under SD; therefore, LD conditions promoted the flowering transition of small bulbs more than cold exposure (Figure 1A). In addition, there was no obvious difference in the plant height and the number of internodes among different weeks of cold storage in small bulbs grown under LD (Figure 1B,C); however, the plant height and internode number under SD conditions were higher than those under LD conditions (Figure 1B,C). For the flower number, all small bulb plants had one flower (Figure 1D).

For the large bulbs planted under SD conditions, the time from planting to VB appearance was inversely proportional to the cold-storage time. When the time of cold-storage increased, the time from planting to VB appearance decreased from 11 weeks to 5 weeks (Figure 1A); at the same time, the flowering rate increased from 32.7% to 83% (Table 1). For the large bulbs planted under LD conditions, with the increase in cold storage time, the weeks from planting to VB appearance decreased from 7 weeks to 4 weeks (Figure 1A), and the flowering rate increased from 48.3% to 100% (Table 1). For the large bulbs, compared to plants grown under SD, LD conditions increased the flowering rate of the nonvernalized large bulb group by 15.6% (48.3−32.7%) and the 9-week cold exposed large bulb group by 17% (100−83%). At the same time, compared with the nonvernalized small bulbs, 9 weeks of cold exposure for large bulbs increased flowering rates by 50.3% (83−32.7%) under SD and 51.7% (100−48.3%) under LD conditions (Table 1). It should be noted that the flower number per plant of large bulbs could increase with increasing cold storage time and LD conditions (Figure 1D).

### 2.2. Expression of Vernalization Related Genes in Scales during Cold Exposure in Lilium Longiflorum

**Figure 2:**
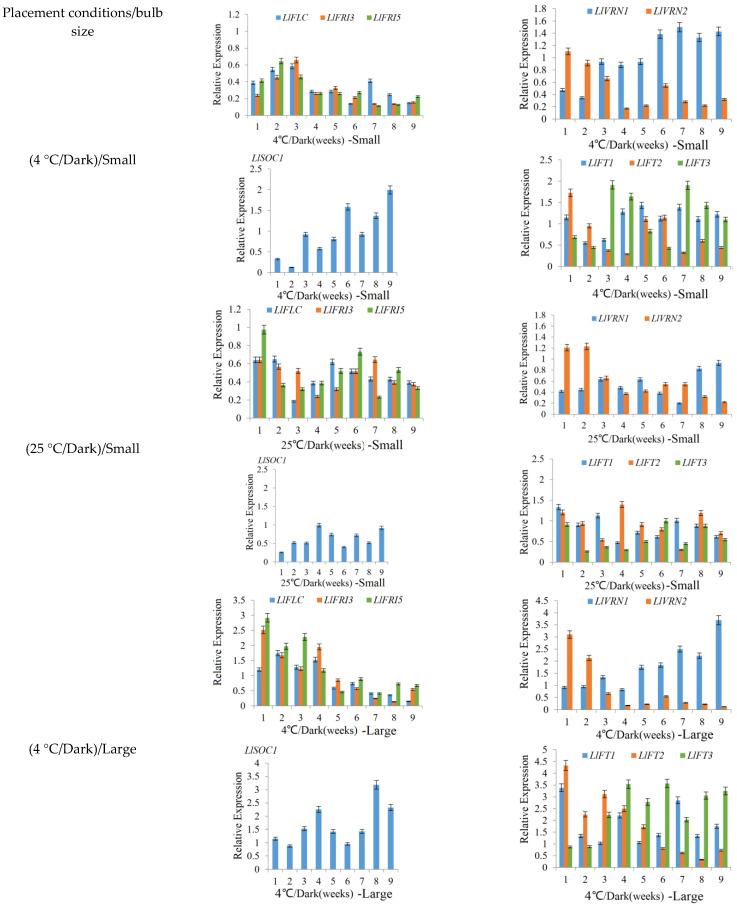

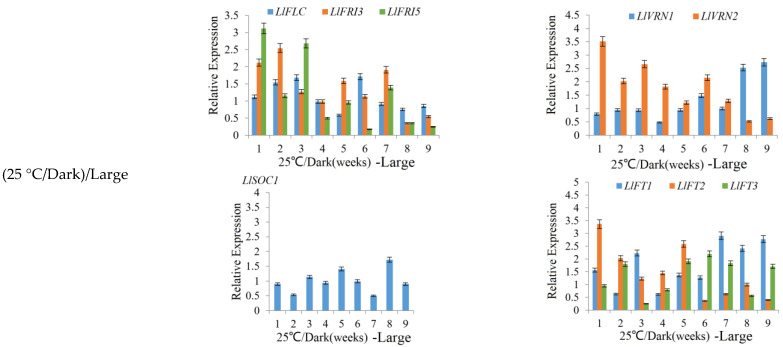
The expression patterns of *LlFLC*, *LlFRI3*, *LlFRI5*, *LlVRN1*, *LlVRN2*, *LlSOC1* and *LlFTs* in scales of *Lilium longiflorum* before planting. Notes: Small and large meant bulb size. Relative expression levels were determined by qRT-PCR (y-axis). Data represented an average of three biological replicates with three technical replicates. Error bars represented SDEV of three biological replicates. The relative expression levels were calculated by the standard E^−ΔΔCq^ method.

The expression patterns of *LlFLC*, *LlFRI3*, *LlFRI5*, *LlVRN1*, *LlVRN2*, *LlSOC1*, *LlFT1*, *LlFT2* and *LlFT3* in scales during cold exposure are shown in Figure 2. The expression levels of *LlFLC*, *LlFRI3*, and *LlFRI5* in both small and large bulbs were significantly decreased during the process of vernalization (4 °C/dark), and the decrease in expression levels in large bulbs was more obvious (Figure 2). For bulbs exposed to 25 °C/dark condition, the expression levels of *LlFLC*, *LlFRI3*, and *LlFRI5* showed a slight downwards trend but no regular changes (Figure 2). However, in all treatment groups, the expression of *LlVRN1* and *LlVRN2* showed opposite trends, and the expression of *LlVRN2* gradually decreased, while the expression of *LlVRN1* showed a significant upwards trend (Figure 2). Among them, the expression of *LlVRN1* in the cold exposed large bulb group increased the most (Figure 2). The expression of *LlSOC1* in the 25 °C /dark treatment groups showed no obvious trends (Figure 2). At the same time, the expression of *LlSOC1*, *LlFT1* and *LlFT3* in both small and large bulbs constantly increased during the process of cold exposure (4 °C/dark). Moreover, it should be noted that the expression of *LlFT3* increased significantly from 3 weeks during the process of vernalization (4 °C/dark), especially in the large bulbs (Figure 2). In the 25 °C /dark treatment groups, there was an irregular change in the small bulbs. The expression of *LlFT2* in all groups showed a downwards trend, and the decrease was more obvious in the large bulbs and the cold exposure (4 °C/dark) group (Figure 2).

### 2.3. Expression of Key Flowering Genes in Leaves of Lilium Longiflorum after Planting

**Figure 3:**
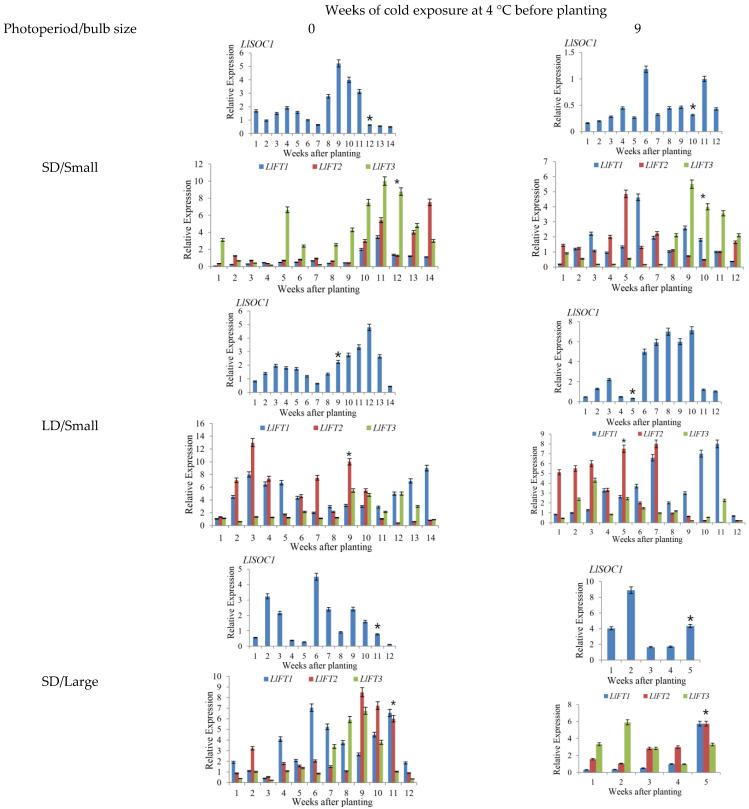

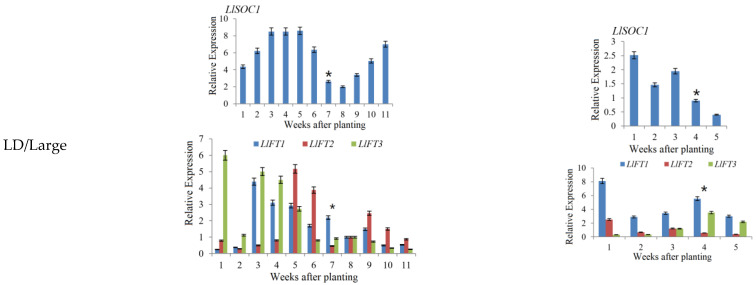
The expression patterns of *LlSOC1*, *LlFTs* in leaves of *Lilium longiflorum* grown under SD/LD conditions. Notes: These abbreviations are the same as above. SD, short day (light/dark: 8/16 h); LD, long day (light/dark: 16/8 h). * visible buds appeared time after planting.

**Figure 4:**
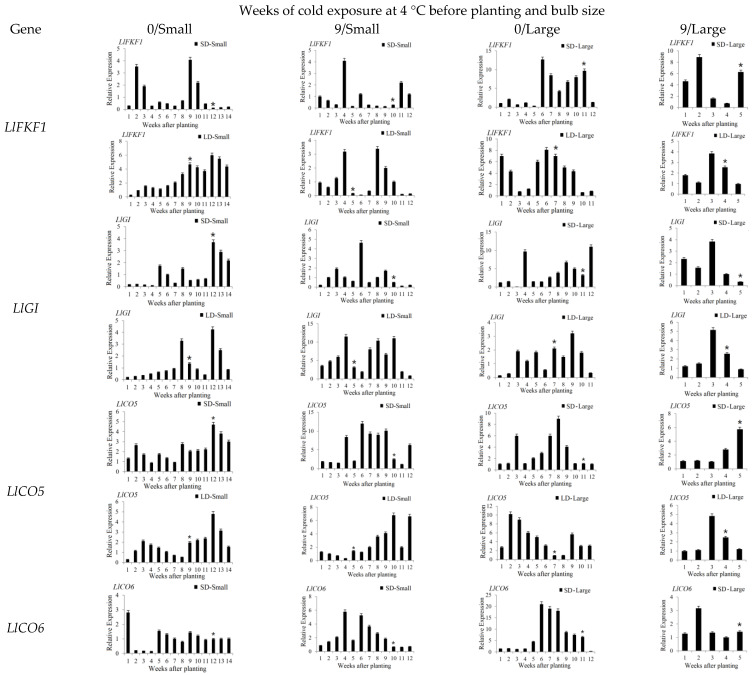

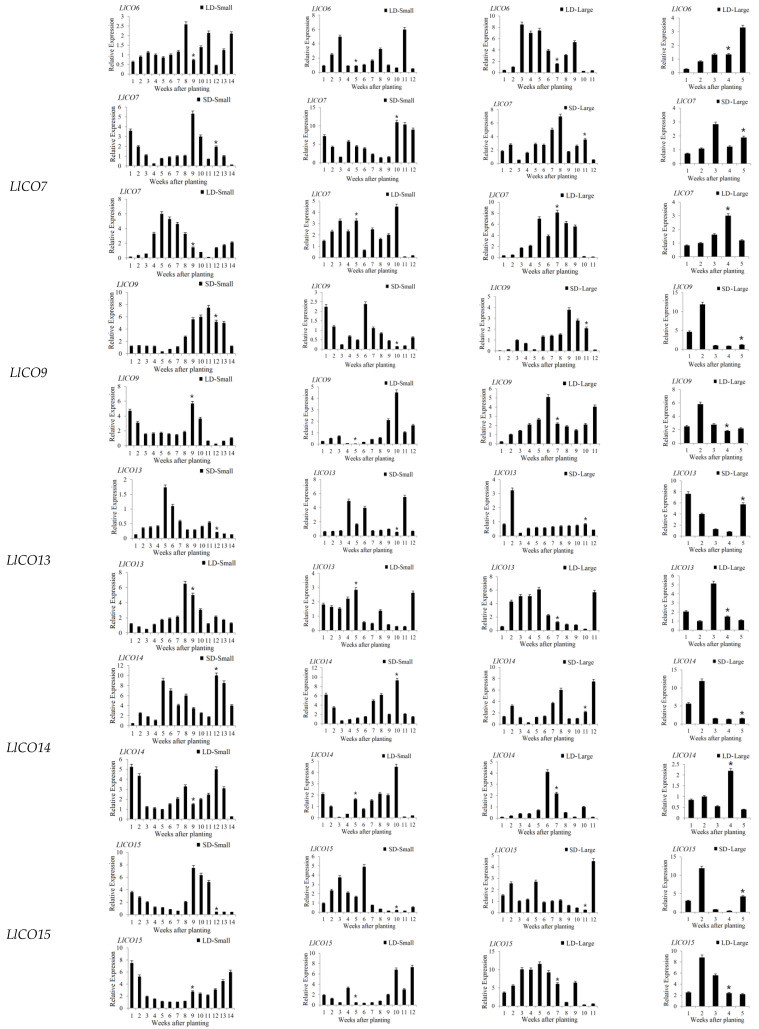

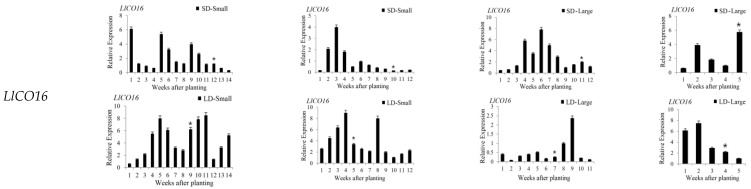
The expression patterns of *LlFKF1*, *LlGI* and *LlCOLs* in leaves of *Lilium longiflorum* grown under SD/LD conditions. Notes: These abbreviations are the same as above. * visible buds appeared time after planting.

To gain insights into the expression patterns of key flowering genes in *Lilium longiflorum*, the expression patterns of *LlCOLs* and key flowering genes in leaves grown under SD/LD conditions were measured for cold exposed (4 °C/Dark/9 weeks) and nonvernalized (25 °C/Dark/9 weeks) large and small bulbs (Figure 3 and Figure 4). According to our previous study [13,14], the critical point of floral transition for *Lilium* was accompanied by an increase in *LFT1* or *LFT3* expression, and *LFT1* may be associated with photoperiod, while *LFT3* may be associated with vernalization [14]. For the cold exposed (4 °C/Dark/9 weeks) large bulb groups, the time from planting to VB under LD and SD conditions was 4 weeks and 5 weeks, respectively. The LD condition had no obvious effect on flowering time, and the expression of *LlFT3* increased significantly from 3 weeks during the process of vernalization. The results indicate that the cold exposed (4 °C/Dark/9 weeks) large bulbs had completed the floral transition before planting. However, for the small bulbs, the time from planting to VB of the cold-exposed (4 °C/Dark/9 weeks) group grown under LD and SD conditions was 5 weeks and 10 weeks, respectively, and the LD condition greatly shortened the flowering time; therefore, the cold-exposed (4 °C/Dark/9 weeks) small bulbs entered the floral transition stage after planting. Moreover, the nonvernalized (25 °C/Dark/9 weeks) small and large bulbs, both completed the floral transition after planting. On these grounds, the increased expression of *LlFT1* or *LlFT3* before visible bud (VB) appearance was considered the floral transition stage of the small bulb and nonvernalized (25 °C/Dark/9 weeks) large bulb group.

According to previous research results, LD conditions played an important role in the flowering of both nonvernalized and cold-exposed small bulbs. For the nonvernalized (25 °C/Dark/9 weeks) small bulbs, under LD conditions, the expression of *LlFT1*, *LlSOC1*, *LlFKF1*, *LlGI*, *LlCO5*, *LlCO6*, *LlCO7*, *LlCO9*, *LlCO13*, *LlCO14* and *LlCO16* was high during the process of flowering transition (Figure 3 and Figure 4). Among them, the expression levels of *LlCO7*, *LlCO13* and *LlCO16* were particularly high before and during the VB stage. However, the expression levels of *LlCO9*, *LlCO14* and *LlCO15* were high in the vegetative stage (Figure 4). For cold-exposed (4 °C/Dark/9 weeks) small bulbs, the expression of *LlFT1*, *LlFT3*, *LlSOC1*, *LlFKF1*, *LlGI*, *LlCO6*, *LlCO7*, *LlCO13* and *LlCO16* was upregulated under LD conditions at the flowering transition stage (Figure 3 and Figure 4). For the *LlCOL* family members, except for *LlCO6*, the others showed consistent expression with that in the nonvernalized small bulbs (Figure 4).

In the floral transition stage of nonvernalized (25 °C/Dark/9 weeks) large bulb group grown under LD conditions, *LlFT1*, *LlFT3*, *LlSOC1*, *LlFKF1*, *LlGI*, *LlCO5*, *LlCO6*, *LlCO7*, *LlCO9*, *LlCO13*, *LlCO14* and *LlCO15* showed increased or high expression before the VB stage (Figure 3 and Figure 4). In addition, the upregulated expression of *LlCO7*, *LlCO9*, *LlCO13* and *LlCO15* was particularly significant before the VB stage (Figure 4).

The expression peaks of *LlFT1*, *LlCO5*, *LlCO7*, *LlCO16* and *LlSOC1* appeared in each group during the floral transition stage and inflorescence stage (Figure 3 and Figure 4). For the small bulbs grown under LDs, the expression of *LlGI* was higher in the cold-exposed treatment group than in the nonvernalized group, and it was speculated that cold exposure may promote greater sensitivity of the small bulbs to LD conditions (Figure 4). *LlCO9* was highly expressed during the flowering induction period in nonvernalized group, and there was no peak expression level at the floral transition stage of cold-exposed plants, so we speculated that its flowering induction function was related to vernalization (Figure 4). The *LlCO13* expression level peaked at the flowering induction stage in the LD group, and its participation in flower formation may be closely related to light (Figure 4). The expression of *LlFT3* was high or peaked in the floral transition stage in the vernalized or SD groups for all sizes of bulbs. The results suggest that *LlFT3* was involved in flowering induction in other flowering pathways, such as the vernalization pathway and autonomic pathway, in addition to the photoperiod pathway (Figure 3).

In addition to the floral induction stage, *LlCO5* was highly expressed in the vegetative stage and the postflowering stage, suggesting that it was involved in the whole growth process of plants. The expression levels of *LlCO13*, *LlCO14* and *LlCO15* were high in the vegetative stage, so it is speculated that these genes are related to the vegetative growth of the plants. Among them, *LlCO15* was highly expressed in each group of *lilium* at the seedling stage (Figure 4). In the early vegetative stage of large bulbs of *Lilium* *longiflorum* plants, *LlCO14* was highly expressed only in the plants that were grown under SD conditions, while it did not have a high expression level under LD conditions, so we speculated that its expression was closely related to the light (Figure 4). The expression of *LlGI*, *LlFKF1* and *LlSOC1* was also high in the vegetative stage and the postflowering stage, suggesting that they were involved in the growth process of plants (Figure 3 and Figure 4). *LlCO6*, *LlCO16* and *LlFT1* showed peak expression levels after anthesis in the LD groups, and they may be involved in the differentiation and formation of small bulblets of plants after the inflorescence stage. The differentiation process of small bulblets may also be closely related to the LD conditions (Figure 3 and Figure 4). The expression peaks of *LlFT2* showed no obvious pattern in different groups at different stages, but it had peaks at the vegetative stage, floral transition stage and postflowering stage, showing that it was widely involved in the whole growth process of *Lilium longiflorum* (Figure 3 and Figure 4).

## 3. Discussion

### 3.1. Vernalization of Lilium Is a Complex Process

Flowering is a very complex process in which plants transition from the vegetative growth stage to the reproductive stage, and flowering is a critical stage for the development of plants. Many factors can affect the floral transition [22,57]. To ensure that the plant blooms under favorable conditions, many plants need to be cold exposed for a period of time, that is vernalization [16,17]. Vernalization is regulated by a series of genes, and the vernalization and flowering processes are greatly affected by the external environmental conditions [1]. In *Arabidopsis*, the key factor in the response to vernalization is *FLC*. *FLC* is a flowering suppressor that mainly encodes a class of MADS proteins. Other genes regulate flower formation by promoting or suppressing *FLC*, and *FLC* can inhibit the genic activity of flowering inducible factors such as *FT* and *SOC1* [15,16]. When plants are under cold exposure at low temperatures, the high expression of *SOC1* and *FT* inhibits the expression of the *FLC* gene and promotes vernalization and flowering in plants. Two other key vernalization genes, *VRN1* and *VRN2*, also play an important role in the vernalization process. Studies in wheat have shown that three *VRN* homologous genes jointly control the response to vernalization. *VRN1* has a certain homology with AP1/Cal/FUL in *Arabidopsis* [58]. *VRN2* mainly encodes a class of TOC1(CCT), CO-like and CO structure Zinc finger proteins and is highly homologous with ZCCT (ZCCT1 and ZCCT2) gene sequences [59]. Moreover, *VRN2* is thought to have the same function as *FLC* as a flowering inhibitor, but it is not evolutionarily relevant. Yan et al. (2006) found that the *VRN3* gene in wheat was highly homologous to *FT* in *Arabidopsis* [19]. According to the results of this study, the flowering of *Lilium longiflorum* with large bulbs was mainly affected by vernalization. It is speculated that *LlFLC* can significantly inhibit the formation of flowers in large bulbs. After vernalization, the expression of *LlFLC* can be rapidly reduced to promote flowering (Figure 2), and the expression of *LlVRN1* increased from the initial stage to the completion of the vernalization process (Figure 2); therefore, *LlVRN1* may inhibit the expression of *LlFLC* to promote flowering. However, the expression of *LlVRN2* showed a similar decreasing trend during cold exposure as that of *LlFLC*, and we speculated that *LlVRN2* could inhibit flowering by promoting the expression of *LlFLC* (Figure 2). Previous studies have shown that the *LoVRN1* gene plays an active role in the response to low-temperature-induced vernalization in *Lilium oriental* hybrids ‘Sorbonne’, *LoVRN1* can promote early flowering in *Arabidopsis*, and the *LoVRN1* gene also plays a role in the change in lily flower type [21]. In addition, when *Lilium* responds to low-temperature conditions, *FLM* (*FLOWERING LOCUS M*) may replace *FLC* to inhibit the expression of the *FT* and *SOC1* genes, thereby regulating the vernalization pathway; however, it is not clear how the *MAF* gene plays a role in the vernalization pathway [21]. Vernalization of *Lilium* is a complex process, and there may be other unique molecular regulatory mechanisms to complete vernalization in *Lilium* in addition to the similar regulatory mode of vernalization as that in *Arabidopsis*.

### 3.2. The Functions of COL Homologous Gene Family Members in Lilium Are Different

**Figure 5:**
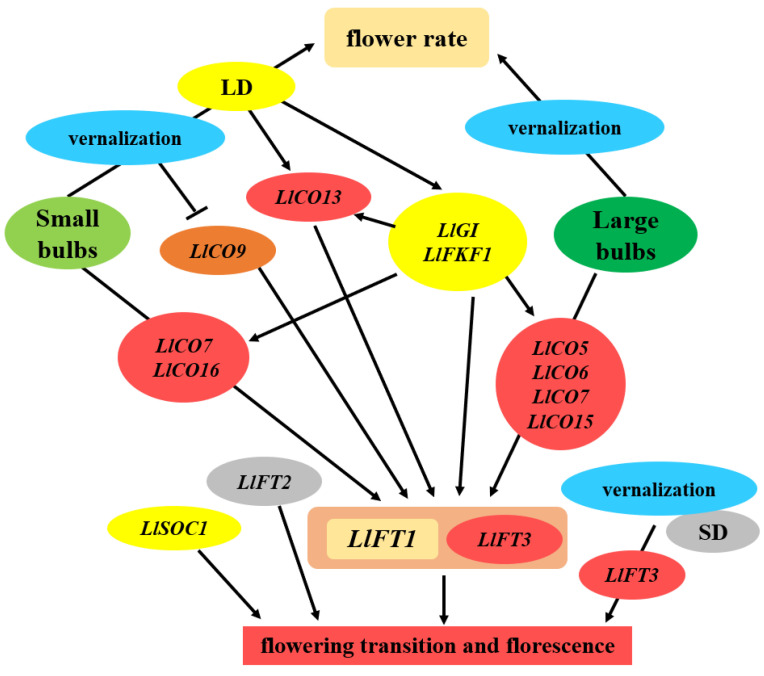
Photoperiod related key gene network in small and large bulbs of *Lilium longiflorum* after planting. Notes: These abbreviations are the same as above. Arrows indicate a promoting, T-ends indicate an inhibiting interaction.

It has been reported that photoperiod induced flowering and bolting in plants often involve members of the *COL* gene family in multiple species, including dicotyledonous and monocotyledonous plants [60]. There are often many members of *COL* gene family in plants, and the number of the *COL* family members varies among different plants. There were 17 *CO* family members in *Arabidopsis*, 16 in *O. sativa*, 12 in soybean, 1 in pea, 3 in tomato and 17 in peanut [27,60,61,62]. In the photoperiod pathway, the *COL* gene can induce the expression of the downstream genes *FT* and *LFY* and then promote plant flowering [5]. The function of *CO* homologous genes in different plants was also different, and some *COL* genes in *Arabidopsis* promoted flowering under LD conditions. The *CO* homologous gene *Hd1* in rice inhibited flowering under LD conditions, but promoted flowering under SD conditions [63,64]. In legumes, ectopic expression of *COL5* could promote the flowering of *Arabidopsis* co-mutants. The overexpression of *GmCOL1a* in soybean led to late flowering, while the *GmCOL1b* mutation promoted flowering [65,66]. Li et al. (2018) obtained eight *LfCOLs* homologous genes from *Lilium* × *formolongi*, and *LfCOL5*, *LfCOL6* and *LfCOL9* were upregulated in the induction phase of flower formation under LD conditions. At the same time, a diurnal expression pattern was found under both LD and SD conditions, and each of these three genes complemented the late-flowering phenotype of the *Arabidopsis* co-mutants and promoted flowering [67]. In this study, *LlCO5* was highly expressed in the flowering induction stage, and may promote the flowering of *Lilium longiflorum* in all groups (Figure 4). Moreover, *LlCO9*′s promotion of flowering was related to vernalization, the expression of *LlCO13* was involved in LD conditions (Figure 5), and *LlCO15* was related to growth in the vegetative stage of *Lilium* (Figure 3 and Figure 4). These results indicated that the functions of *COL* homologous genes in *Lilium* were diverse, and conditions such as cold exposure and LDs could affect the expression patterns and functions of *COL* family members in *Lilium longiflorum*.

### 3.3. The Functions of LFT Gene Family Members in Lilium Are Different

*FT* genes from different species can promote the flowering of *Arabidopsis* [68], and the function of *FT* is generally highly conserved in plants. However, in the evolution of plants, the *FT* homologous genes in different plants have also evolved species-specific functions [69]. In *Arabidopsis*, promoting flowering is the main function of the *FT* gene. Moreover, *FT* also maintains the stability of the flower meristem and promotes inflorescence development, *FT* promotes flowering and thus inhibits vegetative growth of plants [5], and the interaction of *AtFT* and *AtTSF* can regulate lateral branch growth after flowering [70]. *AcFT1* and *AcFT4* in onion promote and inhibit bulb formation, respectively, [11]. SD conditions could promote the expression of *SP6A*, which is an *FT* homologous gene in potato, and thus promote the formation of tubers [71]. *SINGLE-FLOWER TRUSS*(*SFT*) in tomato not only regulates flowering, but also promotes the formation of fruit [72,73]. In previous studies, we found 3 *Lilium FT*(*LFT*) homologous genes in *Lilium*, and the functions of different *LFT* family members were also different [14]. In this study, the expression of *LlFT1* peaked in all groups before the emergence of visible flower buds (Figure 4), which suggested its involvement in the induction of *Lilium* flower formation, consistent with Li’s research results in *Lilium* × *formolongi* [67]. However, *LlFT2* is widely involved in the whole growth process of *Lilium longiflorum*, and *LlFT3* may be involved in floral induction pathways other than the photoperiodic pathway (Figure 3, Figure 4 and Figure 5). These results indicate that the functions of *LFT* gene family members in *Lilium* are different, and the molecular regulatory mechanism between *LFT* and upstream *LCO* gene family members in *Lilium*’s flowering process needs to be further studied.

### 3.4. Vernalization and Photoperiod Significantly Promoted Flower Formation in Lilium Longiflorum

There is a strong interaction between vernalization and the photoperiod, and LD conditions can be equivalent to a 2-week-cold-storage for *Lilium*; that is, there is a ‘2-week effect’. Roh and Wilkins’ study in 1977 showed that LDs could replace cold exposure before planting [43]. Lazare and Zaccais’ research on *Lilium longiflorum* ‘White Heaven’ showed that *Lilium longiflorum* had a high requirement for vernalization, and the nonvernalized large bulbs of *Lilium longiflorum* remained in a vegetative growth state; however, the small bulbs of *L.longiflorum* could blossom under LD conditions with cold exposure. At the same time, vernalization and LD conditions could accelerate the flowering of *Lilium longiflorum* [46]. The results of this study showed that LDs could promote the formation of flowers in *Lilium* with different sized bulbs; among them, small bulbs of *Lilium longiflorum* were more affected by LDs, and large bulbs were mainly affected by vernalization, but all the treatment groups of *Lilium longiflorum* could bloom, including the different sizes of bulbs under SDs and the nonvernalized *Lilium* (Figure 1). These results are different from those of Lazare and Zaccai (2016), who showed that different cultivars of *Lilium longiflorum* need different conditions for flower formation. The ‘White Heaven’ *Longiflorum* hybrid has a greater need for vernalization, but the wild lily *Lilium longiflorum* used in this study may have a weak demand for vernalization. However, these results proved that vernalization and photoperiod significantly promoted flower formation in *Lilium Longiflorum*, and vernalization can significantly promote the flowering of large bulb plants, while LD conditions can significantly promote the flowering of small bulb plants. For *Lilium longiflorum*, both defoliation and lack of light can lead to bud abortions [74,75], indicating that insufficient accumulation of nutrients may block *Lilium* flower formation. In this study, LD conditions greatly promoted the flowering rate of small bulbs (Table 1). Prolonging the cold exposure time of *Lilium* can promote the plant’s growth rate and floral transition [45], and the accumulation of carbohydrates can reduce the proportion of bud abortions [76]; however, the results of this study showed that under SD conditions, increasing the cold exposure time could not promote the flower formation of small bulbs. At the same time, the proportion of large bulbs showing flower formation was significantly higher than that of small bulbs, which also proved the importance of nutrient accumulation for *Lilium* flowering. Some of small bulb plants always stayed at the vegetative growth stage (Figure 1 and Table 1). The size and number of flowers are important for the commercial production of *Lilium* and will affect the quality of flowering in *Lilium oriental* hybrids if the bulb diameter is less than 6 cm [52]. The results of this study showed that all the small bulbs of *Lilium longiflorum* had only one flower, while the number of flowers of large bulb plants was higher than that of small bulb plants; therefore, for the production of *Lilium longiflorum*, if grown in a cold climate where vernalization can occur naturally, using large bulbs for production is better than using small bulbs. However, in warm climates, which usually have LD conditions, the flower formation rate of nonvernalized small bulbs is higher than that of large bulbs, and it is cheaper to use small bulbs for production.

## 4. Materials and Methods

### 4.1. Plants Growth Conditions and Samples Collection

Lily bulbs (‘Qing chun’) were harvested after the aboveground parts withered during autumn (October 2016; temperature average of 25 ± 2 °C), and then bulbs were divided into two groups according to their diameter: 1.5–2.5 cm small bulbs and 4–5 cm large bulbs. All bulbs were placed in moist peat in the dark, the cold exposure groups were placed at 4 °C for 3, 5, 7 and 9 weeks, and the nonvernalized groups were placed at 25 °C for 9 weeks. Samples of central scales (containing the shoot meristem) of bulbs were taken on the first day of every week at 14:00–16:00 before planting. After 9 weeks, all bulbs were planted on the same day and grown in incubators under SD (light/dark:8/16 h) or LD (light/dark: 16/8 h) conditions and a constant ambient temperature of 25 °C. Twenty plants of each group were selected for observation and recording.

During the planting period, the time from planting to visible flower buds, the number of internodes and plant height at visible flower bud, and the average number of flowers were recorded. After all the bulbs were planted, the middle leaves of cold-exposed (4 °C/Dark/9 weeks) and nonvernalized (25 °C/Dark/9 weeks) large and small bulb plants were sampled every week from 16:00 to 18:00, immediately frozen in liquid nitrogen and for further use. All the samples were collected from three biological replicates.

### 4.2. CDNA Preparation and QRT-PCR Analysis

Total RNAs was extracted from the leaves using kits from Aidlab Biotechnology Co., Ltd. (Beijing, China), total RNAs of the scales was extracted with an EasySpin Plus Complex Plant RNA Kit (RN53, Aidlab Biotechnologies Co., Ltd., Beijing, China) according to the manufacturer’s instructions, and then reverse transcribed cDNA with ReverTra Ace qPCR RT Master Mix in conjunction with gDNA Remover (FSQ-301, Toyobo, Japan), according to the manufacturers’ instructions. RNA quality was evaluated by 1% agarose gel electrophoresis and subsequently verified using an ND-1000 spectrophotometer (NanoDrop, Wilmington, DE, USA). The standards applied were 1.8 ≤ OD260/280 ≤ 2.2 and OD260/230 ≥ 1.8.

Specific qRT-PCR primers were designed via Premier 6 on the basis of the coding sequences of *LlVRN1*, *LlFRI3*, *LlFRI5*, *LlFKF1*, *LlGI*, *LlCO5*, *LlCO6*, *LlCO7*, *LlCO9*, *LlCO13*, *LlCO14*, *LlCO15*, *LlCO16*, *LlFT1*, *LlFT2*, *LlFT3* and *LlSOC1* obtained by our research group from the *Lilium* transcriptome, and the specific qRT-PCR primers of *LlFLC* and *LlVRN2* were referred to Liu’ study in 2014 [13,21]. qRT-PCR was performed on a CFX96 Real-time PCR Detection System (Bio-Rad, Hercules, CA, USA) in conjunction with a SYBR Premix EX Taq II Kit (TaKaRa, Beijing, China) according to the manufacturers’ protocols, and the reaction system components were shown in Table S1. The PCR program was as follows: 3 min at 95 °C, followed by 40 cycles of 10 s at 95 °C, 30 s at the annealing temperature, and 15 s at 72 °C, all the samples were run in triplicate in 96-well optical reaction plates. The resulting data were analyzed by CFX Manager software (Bio-Rad). *Lilium* × *formolongi* EF-1a was selected as a reference gene to standardize the results because it was stably expressed at different developmental stages in *Lilium* according to our previous experiment [13]. The sequence information of the primers was listed in Table S2. The relative expression levels in all the figures were measured via qRT-PCR (y-axis), and the data represent the average of three biological replicates, with three technical replicates.

All statistical analyses in this paper were performed using SPSS (Statistical Product and Service Solutions) software.

## 5. Conclusions

The flowering time and rate of large bulbs were greatly influenced by cold exposure, and the vernalization pathway acted more actively at the floral transition stage. The floral transition of small bulbs was affected more by the photoperiod pathway. Moreover, it was speculated that cold exposure may promote greater sensitivity of the small bulbs to LD conditions.

## Figures and Tables

**Table 1:** Flowering rate of small and large bulbs grown under SD/LD conditions.

Group/Proportion of Flowering Plants (%)	Weeks of Cold Exposure at 4 °C before Planting				
	0	3	5	7	9
SD-Small	10	32.33	39	43.22	42.1
LD-Small	52.1	69.21	73.2	78.1	93.25
SD-Large	32.7	37.4	66.9	71	83
LD-Large	48.3	49.1	85.8	100	100

Notes: SD, short day (light/dark: 8/16 h); LD, long day (light/dark: 16/8 h); Small and large, bulb size; 0–9, weeks of bulb exposure to 4 °C before planting.

## Supplementary Materials

The following supporting information can be downloaded at: https://www.mdpi.com/article/10.3390/ijms23158341/s1.
