# Supplementary material for: Expression Patterns of Key Genes in the Photoperiod and Vernalization Flowering Pathways in Lilium longiflorum with Different Bulb Sizes
Source: Int J Mol Sci. 2022 Jul 28;23(15):8341. doi: 10.3390/ijms23158341 (PMC9368551; doi:10.3390/ijms23158341)
Supplement: Supplementary file 1 [file ijms-23-08341-s001.zip › ijms-1765973-supplementary.pdf]

Supplementary material

Table S1 Reaction system of qRT-PCR

Name	Content（μL）
Premix Ex 2×SYBR Premix Ex Taq（RR82LR，Takara，Japan）	10
Upstream primer（10μM）	0.4
Downstream prime（10μM）	0.4
cDNA	2
ddH_2O	7.2
Total volume	20

Table S2 Primers information of *LlFLC, LlFRI3, LlFRI5, LlVRN1, LlVRN2, LlFKF1, LlGI, LlCOLs, LlSOC1, LlFTs* and *EF-1a* for qRT-PCR

Gene	The sequence of primers（**5′→3′**）	Annealing temperature（**℃**）
LlFLC	F: CCTGGTGGAGAAGACGATGAGTG	56
	R: AACAAGATCAGCCGCCAAGTCA	
LlFRI3	F: GTGACTGCATTCCCCGAGAA	55
	R: GAGCAGGATGGCCTTCAACT	
LlFRI5	F: CGATCTCCGCAAAGCTTTCG	56
	R: TCTTGACGCAGAAGGTGACC	
LlVRN1	F: AGTACGGGAGACTGAAGGCT	56
	R: GCTGCTGTCCTCGTATCTCC	
LlVRN2	F: GCTGCCAAACCCTGGTCATCA	57
	R: CATTATGCCTGGTGGTGAGTTCCC	
LlFKF1	F: TGGCACCCCCTTAGTGAATAGA	59
	R: TGCGATGGAAAAAGCCTGAA	
LlGI	F: AACTCCAAGATGGGCTGTCG	56
	R: GAAGCAAAAGGGCAGGAACA	
LlCO5	F: GCGAAGAGGACGGAGATTGA	57
	R: GGCACAACACCATACCCATT	
LlCO6	F: GCTCGCAGATGATGAATGTGT	59
	R: CTCACGCTCTCGTTCTCCAC	
LlCO7	F: GAATGGAGTGGAGATTGAC	55
	R: ACCTTCCTCACCTCATAC	
LlCO9	F: GCACCTATCACATAACAGAGTCC	56
	R: AGGGTGTGTGGTTATTCATCTTTG	
LlCO13	F: CCTCTGCTCCAACTGCGATT	59
	R: CGTCAAAGCCAACTCCAAGC	
LlCO14	F: CGGAGAAGTGTGAAATGATC	55
	R: TGAGGCAATACAAGCAAG	
LlCO15	F: CAAATCATCAGCGGTAGTCAGG	59
	R: CAAGGGGGTGTGGTTCTTCA	
LlCO16	F: AGGGAGGGAGGCAAAGGTAT	55
	R: TTCATCCGTGGTCGCTTCTC	
LlFT1	F：GGGTGGTATATGGTTCAAA	57
	R：CAGTGTAAGTATTCTCTAAGGT	
LlFT2	F：GCATTGGTTGGTGACAGA	57
	R：AGGCGAAGTCTCTAGTGTTA	
LlFT3	F：CAACGATCTCAGGACCGTCT	60
	R：GAGCTGCTGGAACAACACAA	
LlSOC1	F：CGGGCAATCTCTGCGAGTTCTC	57
	R：CAGATTTTCACACAACCACATCCT	
EF-1a	F：GGCACTAACTCGCTCCTTCTG	57
	R：TTGGTAAGATGCTGGTGATTGGAT	
